# Experiences of occupational health doctors and nurses about the role of physiotherapists in occupational health rehabilitation: A qualitative study

**DOI:** 10.1142/S1013702520500018

**Published:** 2019-09-13

**Authors:** Laran Chetty

**Affiliations:** 1 Royal Free London NHS Foundation Trust, Pond Street, London, UK; laranchetty@gmail.com

**Keywords:** Physiotherapy, occupational health, rehabilitation, role, experiences

## Abstract

**Background:**

Occupational health physiotherapy has been practiced in the UK over several decades. In the past decade, the role of occupational health physiotherapy has gained recognition as a profession that can be embedded within occupational health departments; however, limited information is known about the role of physiotherapists from professional groups outside the allied health domain in this context.

**Objective:**

The aim of this study is to explore the experiences of occupational health doctors and nurses about the role of physiotherapy in occupational health rehabilitation.

**Methods:**

This study is a qualitative investigation underpinned by an interpretative construct. Thirteen semi-structured interviews were conducted. Two occupational health doctors and 12 nurses were purposively recruited from two National Health Service (NHS) hospitals. Data were analyzed using thematic content analysis, coded manually and verified by member checking.

**Results:**

The benefits of occupational health physiotherapists were rapid access intervention, advanced knowledge and clinical reasoning, evidence-based practice, and providing an additional perspective. The emerging themes of the challenges that occupational health physiotherapists may face include dealing with occupational health challenges, managing role conflicts, personal qualities and attributes, and role substitution.

**Conclusion:**

Participants described numerous roles of occupational health physiotherapists ranging from clinical to organizational components. On-going research is needed to support the role development of physiotherapists providing occupational health rehabilitation and to further advocate for its relevance in this setting.

## Introduction

Occupational health physiotherapy as a distinct discipline has been practiced in the United Kingdom since the 1940s.^1^ Occupational health physiotherapists are often described as clinicians dedicated to managing employees either autonomously or in conjunction with other members of the team.^2^ Traditionally, occupational health physiotherapy falls under the umbrella of musculoskeletal physiotherapy as an advanced practitioner status due to the specialist knowledge and experience required in this specialty. However, the ‘advanced’ roles in which physiotherapists practice in the occupational health setting is generally poorly documented in the literature. Furthermore, there is no evidence about the experiences of occupational health doctors and nurses with the contribution of physiotherapy to occupational health rehabilitation.^3^

Occupational health doctors and nurses are core professions within the occupational health team and their specialist roles involve reducing the incidence of diseases and injuries, alleviating suffering and promoting and protecting the health and well-being of people in the workplace.^4^ Their experiences with occupational health physiotherapy may reflect different insights of the role and could be used to help guide and market it among members of the occupational health team and to clients and commissioners. Their insights could also aid the acceptability of physiotherapy practice in occupational health rehabilitation where there is resistance and guide future training of physiotherapists working in occupational health rehabilitation.

The aim of this study was therefore to explore the experiences of occupational health doctors and nurses about the role of physiotherapists in occupational health rehabilitation.

## Methods

### Study design

This study used a qualitative framework and was underpinned by an interpretative construct.^5,6^ The qualitative approach allows the researcher to explore in-depth the experiences of occupational health doctors and nurses about the role of physiotherapists providing occupational health rehabilitation at two National Health Service (NHS) hospitals in the United Kingdom.^5^ The interpretative construct allowed for meaningful engagement and dialogue with occupational health doctors and nurses and provides a unique opportunity to gather new insights about how they viewed the role of physiotherapists in this setting.^6^

### The selection of study sites

This study was undertaken at two NHS hospitals of which both offered in-house occupational health services. The two NHS hospitals were strategically chosen because the researcher is not employed by these hospitals and does not line manage any member of the occupational health team. This eliminates the effects of coercion and conflicts of interest and provides the researcher with an outsider’s perspective. The outsider’s perspective is advantageous because the qualitative researcher should preferably enter the research setting as a stranger so that the setting can be viewed with greater insight and more sensitivity not having been decreased by familiarity.^7^ However, an outsider needs to take more time to establish trust with participants which may delay the research.^7^

These two NHS hospitals are comparable in terms of size, bed availability, number of staff employed and patient throughput. Both also feature similar services and have the same structural problems in that they have a combination of century-old buildings and new buildings. Each NHS hospital serves a very different population, with one situated in an affluent area serving a largely homogenous population while the other serving a more culturally diverse population and is situated in a relatively deprived area.

### Participants

Permission was sought from the occupational health manager at each NHS hospital in order to inform them of the study and to gain access to the research sites and recruit participants. A date and time to attend one of their team meetings was agreed with each manager. At each team meeting, the researcher met with members of the occupational health team to explain the details of the study and to hand out study packs to participants. Purposive sampling was used to recruit participants to allow for the selection of only those participants that were considered suitable to the study. The recruitment process involved taking into account the professional group and those with at least three years of occupational health experience. Each study pack consisted of an information sheet, consent form and a prepaid return envelope. Study packs were left with each manager to hand to those participants that were deemed as potentially suitable for the study but were not present at the team meeting. Participants were excluded if they were unwilling or unable, for any reason, to give their written consent. Two occupational health doctors and 12 nurses returned their signed consent form and were included in the study.

### Data collection

Data were collected using semi-structured interviews. Each participant was interviewed in a confidential room onsite. The duration of each interview lasted up to an hour. At the start of the interview, the researcher confirmed if the participant was still willing to take part in the study. The researcher also explained the purpose of the study and reassured participants that all information gathered during the interview would be handled confidentially. In addition, participants were informed that the interviews will be audiotape recorded to permit data analysis at a later point. The interview questions were open-ended to allow for fundamental lines of enquiry relevant to the topic to be pursued with each participant, while also allowing participants the flexibility to freely expand on questions.^6^

In order to gather in-depth insights of the role of physiotherapists in occupational health rehabilitation, the following questions and prompts were formulated: (1) Can you tell me about your experiences with the physiotherapist who provides occupational health rehabilitation? (Prompts: knowledge, behaviors and skills; differences from general outpatient physiotherapy); (2) What kind of services do you expect a physiotherapist in occupational health to offer you? (Prompts: areas of practice; clinical skills; organizational responsibilities); (3) What do you expect physiotherapists in occupational health to offer occupational health services? (Prompts: expert opinion; new/innovative ways of working; on team, clients, managers and outcomes).

### Data analysis

Interviews were transcribed verbatim by the researcher and then independently transcribed by another researcher to ensure accuracy of transcription. Any discrepancies in transcription were resolved by discussion. Thematic content analysis was used to analyze the data. Content analysis is a process of identifying, coding and categorizing the primary patterns of data.^7^ The transcriptions were carefully and repeatedly read, with initial ideas being noted. A list of all ideas was made and similar topics were coded and grouped together to form the main themes. Topics not forming part of the main themes were refined into relevant sub-themes. Quotes from the original transcript were ‘lifted’ and arranged under the relevant sub-theme. A second reviewer independently reviewed the data and themes and any discrepancies in interpretation were resolved by discussion. The second reviewer was not inhibited by closeness to the study and therefore was able to view the data with real detachment and provide a fresh perspective.^8^

### Trustworthiness of the study

Rigor was ensured through credibility, transferability, dependability and confirmability. The researcher built trust by explaining to participants the purpose of the study, utilization and dissemination of the information. Regular notes were kept in a diary to reflect on any emerging assumptions. Thick descriptions were used to enable readers to compare the inferences in the data with those they have seen in their own situation and determine how far they can be confident in transferring to their situation the findings of the study. A detailed description of the study’s operational details was provided. The study was described in as much detail as possible to form an audit trail so that readers could trace step-by-step the decisions made. Finally, data trustworthiness was established through a process of member checking whereby all participants were sent their interview transcripts for review and allowed to make modifications prior to data analysis to ensure its authenticity. No participant asked for any changes or modifications to be made to their transcripts.

### Ethical approval

Ethical clearance was obtained from Middlesex University London Health and Social Care Ethics Committee (Reference: MH35). This study did not require NHS ethical review under the terms of the Governance Arrangements for Research Ethics Committees (A Harmonized Edition) (Reference: 16/SS/0043).

## Findings

The characteristics of participants are presented in Table 1. Participants discussed several components about the role of physiotherapists in occupational health rehabilitation. A list of the themes and sub-themes that emerged is presented in Table 2.

**Table 1.** Characteristics of participants.

Site	Occupation	Gender	Experience	Employment status
Case 1	OH Doctor	Male	25 years	Part-time
	OH Nurse	Female	16 years	Part-time
	OH Nurse	Female	8 years	Full-time
	OH Nurse	Female	4 years	Part-time
	OH Nurse	Female	5 years	Full-time
	OH Nurse	Female	12 years	Full-time
	OH Nurse	Female	3 years	Full-time
	OH Nurse	Male	3 years	Full-time
	OH Nurse	Female	6 years	Full-time
Case 2	OH Doctor	Female	14 years	Part-time
	OH Nurse	Female	13 years	Full-time
	OH Nurse	Male	8 years	Full-time
	OH Nurse	Female	17 years	Full-time
	OH Nurse	Female	12 years	Full-time

**Table 2.** List of themes and sub-themes.

Theme 1:	Benefits of occupational health physiotherapy
Sub-themes:	^∘^ Rapid access intervention
	^∘^ Advanced knowledge and clinical reasoning
	^∘^ Evidence-based practice
	^∘^ Providing an additional perspective
Theme 2:	Challenges of occupational health physiotherapy
Sub-themes:	^∘^ Dealing with occupational health challenges
	^∘^ Managing role conflict
	^∘^ Personal qualities and attributes
	^∘^ Role substitution

## Theme 1: Benefits of Occupational Health Physiotherapy

The four sub-themes under this theme are: (1) rapid access intervention; (2) advanced knowledge and clinical reasoning; (3) evidence-based practice; and (4) providing an additional perspective.

### Rapid access intervention

Participants recognized that early contact with physiotherapists could yield benefits:

“*The fact that we have an occupational health physio on-site helps us manage cases much faster, especially those that come in with acute injuries.*” (Case 1, OH Nurse 2).

“*I would like to have physios in occupational health, especially with all the injuries coming in. It would be really nice if we could have access to physios sooner.*” (Case 2, OH Nurse 3).

### Advanced knowledge and clinical reasoning

Physiotherapists have advanced levels of knowledge and clinical reasoning in complex cases:

“*Physiotherapists provide high-quality and systematic assessments and interventions. We call on them to problem solve complex cases, especially when we cannot sometimes make a decision ourselves.*” (Case 1, OH Doctor).

“*We don’t expect the physiotherapist to provide a generalist role. We have an occupational health physio in the department because they have very specific knowledge, so you get to tap into that knowledge to get a better idea of how injured the employee really is.*” (Case 1, OH Nurse 5).

Occupational health physiotherapists provide more specialized information compared to occupational health doctors and nurses:

“*Sometimes, as a nurse, we are unable to provide the level of detail the employer wants. We tend to give only general advice, like for back pain we say keep active and don’t do any heavy manual handling work, then the employer says that the staff member is already doing this and they want more specific advice. I think there is a need for having physios in occupational health departments who are better placed to deal with these types of cases.*” (Case 2, OH Nurse 1).

“*They are able to evaluate in such detail the effectiveness of interventions, so they are best placed to provide an accurate picture and opinion about how to reduce work injuries.*” (Case 1, OH Nurse 7).

### Evidence-based practice

Participants felt that physiotherapists were better able to refine and implement evidence-based protocols:

“*I think physios are often better at simplifying the evidence and the general consensus is that they tend to use it more often.*” (Case 1, OH Doctor).

“*Physios tend to follow protocols, so they don’t miss anything. I guess they are keen for everyone to be treated according to a standard.*” (Case 1, OH Nurse 6).

One occupational health doctor viewed their workload as being too high and unpredictable to follow guidelines strictly. They felt that physiotherapists had more time to offer dedicated treatment according to evidence-based protocols:

“*A lot of the time, clients prefer to see the physio because they know what to say and do at week 1 of the injury and then week 2 and then a few weeks later. If I see the same client, they don’t get the same type of advice* … *we doctors tend to give the same advice* … *we are probably not as consistent in our advice as physiotherapists.*” (Case 2, OH Doctor).

Occupational health nurses were of the view that physiotherapists not only improve the quality of care provided to clients, but also provide an evidence-based influence on the organization:

“*The physio does not only provide care to our clients, they also deal with issues within the organisation. I think it’s very important they get involved at this level because they have all this knowledge about anatomy and physiology and they can justify why we say what we say* … *if that makes sense.*” (Case 1, OH Nurse 1).

“*I suppose that physios are more involved than we are in assessing because they understand things like human function. We would probably just give clients a back booklet, whereas the physio would know what the latest information is and how to translate this into organisational requirements. We sort of get the ball rolling* … *they are the ones with all the fancy interventions.*” (Case 2, OH Nurse 2).

### Providing an additional perspective

Participants expected physiotherapists to provide an additional perspective in selected occupational health cases:

*“It is better to have physios because they can offer more expertise, which I find compliments the doctor’s advice.”* (Case 1, OH Nurse 4).

*“I think the more specialists there are on the team to assist staff with all sorts of conditions, the better* … *this will ensure that staff get better care, so I think physiotherapists can help make care better*.” (Case 2, OH Nurse 1).

Physiotherapists were also expected to work in collaboration with occupational health doctors and nurses and not in isolation:

*“Physiotherapists would need to liaise directly with doctors and nurses if they want to offer a different opinion so that any disagreement can be resolved and the best advice is given to staff*.” (Case 1, OH Nurse 3).

*“There is no doubt in my mind that physiotherapists have unique skills, so having them around makes the occupational health service more complete because they can supply more input into the cases, which the doctors might not have thought about*.” (Case 1, OH Nurse 4).

## Theme 2: Challenges of Occupational Health Physiotherapy

The four sub-themes under this theme are: (1) dealing with occupational health challenges; (2) managing role conflicts; (3) personal qualities and attributes; and (4) role substitution.

### Dealing with occupational health challenges

Participants agreed that occupational health departments’ deal with many challenges and physiotherapists had a crucial role in helping to alleviate some of these challenges, such as the long waiting times and limited departmental resources:

*“One of the crucial issues in occupational health is the waiting times* … *and the nurses are so busy with other things* … *they don’t always have the time to deal with all of this. I think this is where the physiotherapist comes in* … *helping to reduce the wait to be seen*.” (Case 1, OH Nurse 2).

Another challenge was the lack of specialized clinicians dealing with certain cases and the multiple problems presenting to occupational health departments:

…*“especially for the acute musculoskeletal cases, I don’t think the nurses and even the doctors are skilled enough to deal with some of these. The physiotherapist can help with early management to resolve these injuries*.” (Case 1, OH Nurse 4).

### Managing role conflicts

Some occupational health doctors and nurses viewed the advancement of physiotherapy’s role in occupational health departments as a potential threat. Some occupational doctors and nurses were concerned that an advancing physiotherapy role could make it more difficult for them to justify their own positions:

*“There seems to be no structure these days about who does what* … *our roles seems to be getting blurred all the time, first with nurse-led services and now with the addition of physiotherapists. It’s very difficult to say I’m a consultant and I do this because the physios and the nurses do it as well*.” (Case 2, OH Doctor).

*“I think if doctors do their bit, nurses do their bit and similarly physios do their bit, and we all work closely together, then it really works well. I think it’s only a problem when some professions try to go beyond what they are trained to do*.” (Case 2, OH Nurse 1).

Although choosing the best candidate for a role is logical, the general view of the physiotherapy role was that it would suit someone who was aware that their role was constantly under scrutiny by other professions within the team and that they had to constantly clarify their position within the team:

*“As an occupational health nurse I have to constantly clarify my position and show the value and attributes I bring to the post. Occupational health physiotherapists are not traditional members of the team, and so it is easy to get a bit confused about their special traits*.” (Case 1, OH Nurse 8).

### Personal qualities and attributes

There were certain professional and personal attributes that physiotherapists are required to possess. This involved being able to competently perform a range of physiotherapeutic treatment modalities, have good time management and can demonstrate conflict resolution skills. These attributes were even part of the recruitment process:

*“While it is important to get someone with a range of skills, I think it is also necessary to get someone that has the attributes to cope with the demands of the job and be able to deal with difficult managers*.” (Case 1, OH Nurse 5).

### Role substitution

Physiotherapists were also required to occasionally substitute for the role of an occupational health doctor or nurse as they acknowledged that physiotherapists had similar skills and knowledge:

*“We had a client who was having trouble with his hip and he didn’t need to see the doctor because the physio could assess and tell his manager he could come back to work and what should be avoided. So he really didn’t need to see the doctor*.” (Case 1, OH Nurse 2).

*“I see no reason why a physiotherapist cannot reassure staff and tell them how to deal with their injuries. Clients don’t need to wait for the nurse or doctor*.” (Case 2, OH Nurse 4).

## Discussion

The aim of this study was to explore the experiences of occupational health doctors and nurses about the role of physiotherapists providing occupational health rehabilitation. A higher number of nurses participated in this study which is in keeping with the higher number of nurses employed in occupational health departments. The semi-structured interviews were designed in a way that all participants were asked similar questions while allowing for more in-depth probing to cover a wide range of topics about the role of physiotherapists providing occupational health rehabilitation.

Occupational health doctors and nurses reported several benefits of physiotherapists providing occupational health rehabilitation. In particular, rapid access to physiotherapists was perceived as beneficial to clients attending an occupational health service so that they do not have to wait in long queues for access to primary care physiotherapy. Furthermore, rapid access to physiotherapy services is a national occupational health service quality requirement^8^ and physiotherapists should be mindful that occupational health doctors and nurses expect clients to have early access to their services to avoid being perceived as providing an inefficient service. A study by Addley *et al.* on the benefits of a rapid access physiotherapy service in an occupational health setting found significant improvements in health outcomes and enabled those absent from work to return to work earlier.^9^

The advanced level of knowledge and clinical reasoning of physiotherapists in occupational health, beyond that of a generalist physiotherapist, was perceived as an essential component of occupational health physiotherapy practice. This advanced level of knowledge and reasoning is required because physiotherapists in occupational health must provide an expert opinion on both clinical and organizational issues. One of the most effective means of reducing resistance to a physiotherapy role and showing it can make a difference is demonstrating clinical effectiveness.^10^ Some of the occupational health clinicians interviewed accepted they had limited knowledge with regards to musculoskeletal injuries and it could be argued that physiotherapists add value to the occupational health service by providing advanced knowledge and reasoning in the rehabilitation of these injuries.

The ability of physiotherapists to provide an additional perspective within an occupational health department was seen as an important role to help filter the referrals coming into the service. This would involve identifying those that are at high risk, those with complex injuries and may have difficulty performing their job, and those that are potentially at risk of sustaining injuries. Arguably, one of the most important contributions that physiotherapists can make to an occupational health department is providing appropriate advice following a referral in order to avoid inappropriate use of occupational health doctors and nurses’ time, in particular occupational health doctors, to focus on complex medical cases. Phillips *et al.* evaluated the cost-effectiveness of physiotherapy support for NHS occupational health services and found that physiotherapists were not only skilled to deal with a range of musculoskeletal disorders of the back, neck and upper and lower limbs, but this service had a cost benefit which represents value for money.^11^

Occupational health doctors and nurses also reported challenges that physiotherapists may experience when providing occupational health rehabilitation. Participants viewed the occupational health department as a complex working environment that is influenced not only by clinical care, but by demanding occupational health challenges and organizational changes. In order to address these challenges, participants felt that physiotherapists must be able to balance their clinical role while meeting organizational needs and be able to deal with the presenting occupational health challenges. This will enhance the influence and effectiveness of occupational health physiotherapists on decision makers.^12^

Participants anticipated that physiotherapists who were part of the occupational health department may experience role conflicts with the traditional members of the occupational health team. In the context of this study, role identity is conceptualized as the character people play (that is, the occupational health physiotherapist) when holding specific social positions in groups (that is, the occupational health team).^13^ Furthermore, according to Burke and Stets (2009) it is relational, since people interact with each other via their own role identities. In this regard, the physiotherapist who is part of the occupational health team has the advantage of being constantly visible and easier to access with the traditional members of the team to make certain there is a collective agreement on the role of the physiotherapy and reduce any role conflicts. It will also ensure that the physiotherapist is able to promote health and injury prevention strategies from a rehabilitation perspective.^14^

The experience of participants was that although choosing the most appropriately qualified and experienced candidate for a role is logical, the recruitment of the occupational health physiotherapy role is such that it would suit someone who can demonstrate awareness that their role is constantly under scrutiny by other professions within the team. This involves having to constantly clarify their position in the department, and being able to challenge medical opinions and those of the referring manager, especially when it was contradictory to their own professional recommendations.

It is also vital for physiotherapists to demonstrate an advance level of clinical knowledge and reasoning because this may assist with fostering trust, respect and acceptance in occupational health departments. Some participants felt that it was vital for physiotherapists working in occupational health rehabilitation to demonstrate an advance level of clinical knowledge and reasoning because it may allow them to confidently undertake some of the work traditionally performed by occupational health doctors and nurses. However, there was concern that physiotherapists must receive adequate training to carry out any new components in their role so that they do not risk practicing outside the scope of their knowledge.

There were some participants, however, that were concerned about the advanced clinical role that occupational health physiotherapists were performing, and how it threatened their own roles. Reed *et al.* (2009) did warn that when dealing with different stakeholders, conflicting and diverse agendas would come up and this had to be addressed. This is supported by the literature which recognizes that positive outcomes are at risk if departmental staff do not work together to reduce clinical errors.^15^ Furthermore, according to Atwal and Caldwell (2002), understanding the roles of each other is essential to effectively collaborate on clinical management and avoid duplication of professional roles, waste resources and miss clinical signs in the interest of protecting clinical turf.^16^

The limitation of the study is its generalizability given the restriction of the study to only two NHS hospitals. Although only two cases were used, it is also an example of a broader group, and therefore offers the prospect of transferability in which readers can judge for themselves the applicability of the findings to their own settings and context.

## Conclusion

The analysis of the qualitative data produced sub-themes that can be applied immediately to physiotherapy practice within occupational health departments and can be used to further advocate for its relevance in this setting. From the insights of occupational health doctors and nurses, physiotherapists embedded within the occupational health team is likely to accomplish the key elements of a safe, effective and quality occupational health service. Given the importance of team integration, future research should incorporate various other stakeholders to ascertain the role of physiotherapists within occupational health rehabilitation. This information may then be used to build on the sub-themes generated in this study and can be used to promote awareness of the contribution of physiotherapists in line with the intended direction of occupational health services.

## Acknowledgment

The author would like to thank all participants for taking part in the research study.

## Conflict of Interest

The author has no conflicts of interest to declare.

## Funding/Support

This work was supported by Arthritis Research UK.

## References

[1] Daley D, Miller M Moving forward in occupational health physical therapy: The journey toward specialisation in the United States. Phys Ther Rev 2013;18:316–26.

[2] Chetty L A critical review of physiotherapy as a clinical service in occupational health departments. Workplace Health Saf 2014;62:389–94. 25208353 10.3928/21650799-20140804-01

[3] Chetty L Perceptions of workforce managers about the role and responsibilities of physiotherapists in occupational health rehabilitation: A qualitative study. Prog Med Sci 2017;1(1):14–18.

[4] Nicholson PJ Occupational health services in the UK — challenges and opportunities. Occup Med 2004;54:147–52. 10.1093/occmed/kqg125 15133136

[5] Walsham G Doing interpretive research. Eur J Inf Syst 2006;15:320–30.

[6] Punch K Introduction to social research: Quantitative and qualitative approaches. London: Sage, 2005.

[7] Bonner A, Tolhurst G Insider-outsider perspectives of participant observation. Nurse Res 2002; 9(4):7–19. 10.7748/nr2002.07.9.4.7.c6194 12149898

[8] Safe Effective Quality Occupational Health Service (2015) Introduction to SEQOHS accreditation for OH physiotherapy services. Available from: https://www.physio.seqohs.org/CMS_Documents/Scheme/OH/171005%20%20document%20%20 Introduction%20to%20SEQOHS%20for%20OH%20physiotherapy%20services%20booklet%20V1.2.pdf.

[9] Addley K, Burke C, McQuillan P Impact of a direct access occupational physiotherapy treatment service. Occup Med 2010;60:651–53. 10.1093/occmed/kqq160 20952558

[10] Grimmer K, Beard M, Bell A, Chipchase L, Edwards E, Fulton I, Gill T On the constructs of quality physiotherapy. Aust J Physiother 2000; 46(1):3–7. 11676784 10.1016/s0004-9514(14)60308-1

[11] Phillips CJ, Phillips R, Main CJ, Watson PJ, Davies S, Farr A, Harper C, Noble G, Aylward M, Packman J, Downton M, Hale J The cost effectiveness of NHS physiotherapy support for occupational health services. BMC Musculoskeletal Disorders 2012;13:29–39. 22361319 10.1186/1471-2474-13-29 PMC3382429

[12] Chetty L Effectiveness of physiotherapy provision within an occupational health setting Indian. J Physiother Occup Ther 2011;5(3):50–3.

[13] Burke PJ, Stets JE Identity theory. New York: Oxford University Press, 2009.

[14] Chetty L Occupational health promotion and injury prevention strategies: The rehabilitation perspective. J Commun Health Sci 2011;6(1):60–6.

[15] Reed MS, Graves A, Dandy N, Posthumus H, Hubacek K, Morris J, Prell C, Quinn CH, Stringer C Who’s in and why? A typology of stakeholder analysis methods for natural resource management. J Environ Manage 2009;90:1933–49. 19231064 10.1016/j.jenvman.2009.01.001

[16] Atwal A, Caldwell K Do multidisciplinary integrated care pathways improve interprofessional collaboration? Scand J Caring Sci 2002; 16(4):360–67. 12445105 10.1046/j.1471-6712.2002.00101.x

